# Prevalence of male participation in modern contraceptive use among married men in Durame Town Southern Ethiopia: a community based cross sectional study, 2021

**DOI:** 10.11604/pamj.2022.41.307.32402

**Published:** 2022-04-14

**Authors:** Teketel Ermias Geltore, Yosef Yohannes Lakew

**Affiliations:** 1 Department of Midwifery, College of Medicine and Health Sciences, Wachemo University Durame Campus, Durame, Ethiopia,; 2 Department of Nursing, College of Medicine and Health Sciences, Wachemo University Durame Campus, Durame, Ethiopia

**Keywords:** Contraceptive methods, community based study, Durame Town, men’s involvement, Ethiopia

## Abstract

**Introduction:**

failure to involve men in the family planning programs in a male-controlled society as Ethiopia, has serious consequences even if women are interested to use contraception their husbands oppose them. Therefore, pointing men for contraceptive methods interventions may meaningfully increase contraceptive acceptance by the provision of information, education and communication. Thus, this study assessed prevalence of men participation in family planning utilization in Durame Town, Southern Ethiopia, 2021.

**Methods:**

a community based cross-sectional study was employed. Systematic random sampling technique was used to select study subjects. Data was collected using pretested structured interviewer administered questionnaire. Descriptive analysis was done to describe the study population. Bi-variable and multi-variable logistic regression analyses were done to identify factors associated with participation of men in family planning.

**Results:**

from 382 currently married men who were interviewed, 366 were responded to the questionnaires making the response rate of 96%. The mean age of the participants was 34 (± 6.1) years. Majority of the participants were Kembata 311 (85%) by ethnicity and Protestant 257 (70.2%) by religion. Regarding respondent’s occupation about 153 (41.8%) were merchants and more than half of respondents´ monthly income was >2500 Ethiopian birr. The prevalence of male participation in family planning was 255 (69.7%). Men's participation in family planning was significantly associated with educational, number of currently living children, source of information, knowledge and attitude of the respondents.

**Conclusion:**

strategies and programs aimed at increasing contraceptive prevalence should appropriately address the involvement of males and integrating them use the contraceptive methods.

## Introduction

Family planning denotes to a careful effort by a couple to limit or space the number of kids by contraceptive methods [1]. Family planning is very crucial in terms of decreasing maternal mortality, reducing poverty, environmental degradation and adolescent gestations [2,3]. Husband support is a very important aspect that increases the possibility to use a contraceptive method by women. For instance, in China, husbands´ involvement in the counseling process contributed in reducing rates of unwanted pregnancy [4]. Participation of males in family planning is critical as it positively influences access, uptake and decide a woman´s current or future use of contraception [5].

Indeed, an understanding of men´s perspectives on family planning and reproductive health could provide more insights than are possible by studying women because men have more power than women do in reproductive decision making [6]. Disregard men in fertility programs undermines the need for successful reproductive health indices that is central to achieve global initiatives such as ending preventable child and maternal deaths. It is thus, crucial to put service care providers in the image while designing to stabilize population number by addressing the family life and sexual activities of men [7]. In developing countries, men desire to have many children than their wives. Factors like gender norm, inadequate male involvement and socio-cultural judgments on behaviors of women and men often influence women ability to discuss about contraceptive utilization actively in order to improve their livelihoods. Even if males have knowledge in family planning nationwide, but their involvement is neglected in some areas [6,8].

Male participation in reproductive health services practice incorporates the manner men receive and indicate support to their partners´ interest and rights to use family planning [9]. In many families especially in Africa, many men are not on the way of participating in the planning of the family. Hence, then family planning is considered as women´s business even though men make most of the household decisions including family size [10]. Yet, early marriage and delivering numerous children, without gap is a common practice of low-income countries including Ethiopia. The total fertility rate is 4.6 nationally and 4.4 in Southern Nations Nationalities and People Republic [11]. In a country such as Ethiopia with high fertility rate and unmet need of contraception to ascertain the continuity and sustainability of the service, effective family planning utilization is an important strategy [12-15]. Even though, Ethiopian ministry of health has planned to work on the providing of all family planning (FP) methods, in the lowest service delivery by making free of cost but its utilization is low [11]. If men have inaccurate evidence on the benefits of various family planning methods, they may resist supporting family planning use because of misinformation that some methods may harm the woman´s health [16].

Fundamental agenda of developing countries is to solve their fast and unrestrained increase in population. It is well written that men´s general knowledge and their attitudes, gender preference of children, ideal spacing between child births and contraceptive method use significantly affect women´s choices and views [17]. Participation of men is not only limited to receiving contraceptive restricted methods but also incorporates men who encourage their partners and their friends to use family planning [18]. Couples may have an unmet need for contraception for a variety of explanations: inadequacy of knowledge about unwanted gestation, anxiety about side effects, perceive that their husbands, their religion opposes family planning as well as lack of access to the services [5,10]. In ability to address men participation in contraception in male dominated Ethiopian communities decrease the interest even women are motivated to practice contraception. Thus, according to Ethiopian demographic health survey of 2016 unmet for family planning was 22%. So, in order to overcome such problems, involving men in family planning program is a vital [11]. Thus, this study assessed prevalence of men participation in modern family planning utilization among married couples in Durame Town, Southern Ethiopia, 2021.

## Methods

**Study design:** a community based cross sectional study design was employed from July 1^st^ to 15^th^ in Durame Town in Kembata Tembaro Zone, Southern Ethiopia, 2021.

**Settings:** Durame Town is located in Southern Ethiopia at 340 km from Addis Ababa, the capital city of Ethiopia. According to the 2013 EC Durame the town report, the total population was 52,513, from which 20,138 were males´ and 32,375 were females. According to the town health office, there are three kebeles with 11,460 households and there is one general hospital, two health centers and sixteen health posts.

**Participants:** the source population was all married men in the age group of 20-65 years, who stayed in Durame Town for at least six months in the study area.

**Dependent variables:** male participation in contraceptive methods.

**Independent variables:** socio demographic characteristics, fertility desire and reproductive history, spousal communication, knowledge of the respondents and attitude related factors and institution, community, and policy level related factors.

**Study size:** the sample size for this study was calculated using a single population proportion formula by considering: 95% confidence level, margin of error (0.05), (p=0.655) and by using the formula


$$n=\frac{{\left(Z\alpha /2\right)}^{2}x\text{ p(1-p)}}{d^{2}}$$

It was computed by considering 0.655% from a previous study [19]. The estimated sample size, by using the above-mentioned formula and by considering 10% none response rate thus, the final sample was three hundred eight two. A simple random sampling technique was undertaken to select the Kebeles. The two Kebeles were included in the study. Participants were allocated proportionally to get 382 married males from 6,318 husbands residing in randomly selected two Kebeles. Samples were allocated proportionally to each Kebele based on their total household. The married males were selected systematically and every 16 households in each kebele were included in the survey.

### Operational definitions

**Male participation:** those men who are sharing at least in either of; communication, assist and usage of contraceptive methods by both of them.

**Positive attitude:** based on the statements assessing attitude, the mean score (60%) of the distribution was considered as having positive attitude towards family planning [20].

**Adequate knowledge:** based on the summative score of questions designed to assess knowledge, men with above the mean of the distribution or 60% were considered as having better knowledge of family planning services [20].

**Current use of family planning:** those participants who are using the family planning method by themselves or their partners are using during the data collection period.

**Awareness of family planning methods:** a participant knows at least one modern method of family planning.

**Inclusion criteria and exclusion criteria:** all married men aged 20-65 years old who resided in Durame Town at least for six months. Married men who were not available during data collection period and those who stayed for less than six months were excluded from the survey.

**Data collection procedures and instruments:** data were collected using interviewer administered questionnaire prepared by adapting from different studies [5-9,18]. The tools first were prepared in English, and then converted to Amharic and back to English again to check validity. Five data collectors and two supervisors were assigned. Two days training was given to the data collectors and supervisors, before actual data collection.

**Data management and analysis:** data consistency was checked and entered into EpiData version 3.1 then was exported to Statistical Package for Social Sciences version 20 for further analysis. Tables and figures were used for data presentation. Bivariate logistic regression was used to identify factors associated with prevalence of male involvement in family planning based on OR, 95% CI and p-value of less than 0.25. In multivariable logistic regression model the variables which have independent association with prevalence of male involvement in family planning were identified by considering AOR, with 95% CI and p-value less than 0.05. Data quality was ensured during data collection, coding, entry and analysis. The collected data was checked for completeness by data collectors and supervisors on a daily basis. In this particular study, reliability was assessed by using Cronbach´s alpha to assess the internal consistency of attitude, knowledge and practice questions.

**Ethics approval and consent to participate:** ethical approval for this study was obtained from Research and Community Service Directorate Office, Wachemo University Durame Campus (D/C RCS/590/2013) to conduct the study. Agreement to conduct the study was secured from the officials at different levels including Durame Town municipality, administrative office and selected Kebeles. Oral informed consent was obtained from respondents after giving them information about the study. Participants were informed about the purpose and objective of the study. They were also being told that they would have every right to discontinue to participate in the study.

## Results

From 382 currently married men were interviewed, 366 were responded to the questionnaires making the response rate of 96%. Table 1 shows the socio-demographic characteristics of the participants. The mean age of the participants was 34 (± 6.1) years. Majority of the participants were Kembata 311 (85%) by ethnicity and Protestant 257 (70.2%) by religion. Regarding respondents´ occupation about 153 (41.8%) were merchants and more than half of respondents´ monthly income was >2500 Ethiopian birr.

**Table 1:** the socio-demographic characteristics of currently married men in Durame District, Southern Ethiopia, 2021 (N=366)

Variables	Frequency	Percentage
**Age of respondent**		
20-30	2	0.5
31-40	16	4.4
41-50	166	45.4
51-65	182	49.7
**Ethnicity**		
Kembata	311	85
Hadiya	4	1.1
Amhara	32	8.7
Gurage	6	1.6
Others**	13	3.6
**Religion**		
Protestant	257	70.2
Orthodox	48	13.1
Catholic	53	14.5
Muslim	8	2.2
**Educational status of the respondent**		
Illiterate	36	9.8
Can read and write	91	24.9
Primary education and secondary school	111	30.3
Diploma and above	128	35
**Occupational status of the respondent**		
Governmental employee	136	37.2
Merchant	153	41.8
Daily laborer	47	12.8
Student	30	8.2
**House hold income Ethiopian birr**		
≤ 1199	46	12.5
1200-2499	20	5.5
2500	95	26
> 2500	205	56
**Having radio or TV**		
Yes	328	89.6
No	38	10.4

** Silte, Wolaita

**Figure 1:**
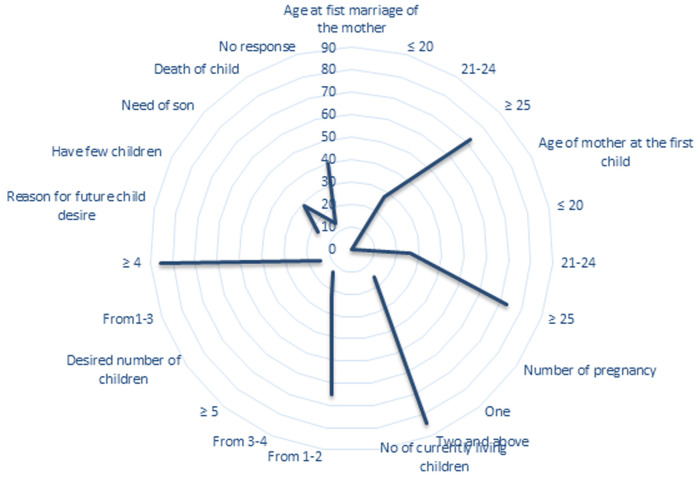
shows fertility desired and reproductive history of respondents in Durame District, Southern Ethiopia, 2021, (N=366)

**Fertility desired and reproductive history of married men:** more than half, 264 (72.1%) of the study participant’s wives were married at the age of ≥25 with the mean age at first marriage of 27.2 (± 4.5). Majority 308 (84.2%) of study men reported that their wives were pregnant for more than two times. More than half (253 (69.1%) of respondents had discussions with their wives on the number of children that they want to have and the majority of the participants (53 (14.5%) wished to have less than or equal to three children, while 313 (85.5%) of the respondents wished to have greater than or equal to four children. About 146 (40%) respondents have no response for future child desire, whereas, 62 (17%) mentioned that they have few children. More than half 283 (65%) of respondents have 1-2 children while 81 (22.2%) and 47 (12.8%) of the study subjects have 3-4 and greater than or equal to five children respectively as shown in Figure 1.

**Support and spousal communication:** the findings shown in Table 2, about 255 (69.7%) respondents were participated on family planning methods. About 65% study subjects support their spouses, 45% of the respondents were discussed about family planning in the last 1 year with their wives to child spacing and/or to limiting birth. During study period, about 55.5% of married couples were using family planning methods which were approved by jointly (75%). On one hand, about 40% of married couples using pills followed by implants (30%). On the other hand, only 9.8% study subjects were directly involved in the use of family planning mostly male condom.

**Table 2:** support and spousal communication in family planning in Durame District, Southern Ethiopia, 2021 (N=366)

Variables	Frequency	Percent
**Male involvement in family planning (n=366)**		
Involved	255	69.7
Not involved	111	30.3
**Have you ever used family planning (n=366)**		
Yes	36	9.8
No	330	90.2
**What is the responsibility of male during family planning (n=20)**		
Support wife	12	60
Allocate budget	1	5
Discuss on the use	5	25
Have no role	2	10
**Have you discussed about family planning in the last 12 months with your wife (n=20)**		
Yes	9	45
No	11	55
**Currently use of family planning (n=36)**		
Stopped	16	44.4
Using	20	55.6
**Purpose of using the methods**		
Birth spacing	300	81.9
Birth limiting	66	18.1
**Whose choice is the current FP (n=20)**		
My wife	5	25
Both of us	15	75
**Types of family planning used (n=20)**		
Implants	6	30
IUCD	3	15
Condoms	2	10
Pills	7	35
Injectable	1	5
Vasectomy	-	-
Bilateral tuba ligation	1	5
**From where do you get the service of family planning service**		
Health institution	69	18.9
Friends	63	17.2
Media	196	53.6
NGO	38	10.3

IUCD: intra-uterine contraceptive device; FP: family planning; NGO: non-governmental organization

**Knowledge of respondents on modern contraceptive methods:** most of the study respondents (95.6%) reported that they had ever heard about FP methods. About 64.5% of respondents listed 2-3 family planning methods. Among those who had ever heard about family planning, the majority about 158 (43.7%) heard through the mass media and 86 (23.5%) from health institutions. About 239 (65.3%) of the respondents have exposure to family planning in the last one year. Based on the composite knowledge score, more than half 201 (54.9%) study subjects had enough knowledge about family planning, while 165 (45.1%) had insufficient knowledge as shown in Table 3.

**Table 3:** knowledge of respondents on modern contraceptive methods in Durame District

Variables		Frequency	Percentage
Hear about family planning	Yes	350	95.6
	No	16	4.4
**List of family planning**			
	One	90	24.5
	Two to three	236	64.5
	Greater than four	40	11
**Source of information**			
	Health institution	86	23.5
	Friends	80	21.9
	Media	160	43.7
	Ngo	40	10.9
Exposure to family planning	Yes	239	65.3
	No	127	34.7

**Attitude of respondents on modern contraceptive methods:** regarding the attitude of the respondents, majority (292 (79.8%) of the study subjects agreed that family planning is important for child spacing. About 279 (76.2%) of the respondents were not sure that insertion and removal of implant is highly painful. Majority 281 (76.8%) of study subjects were not sure that insertion of intra uterine contraceptive device causes lose to secrecy. About 283 (77.3%) of the respondents were not sure that using intra uterine contraceptive device limit usual activities. More than half respondents (236 (64.5%) were not sure that procedure for bilateral tuba ligation is hazardous. Based on the composite attitude score, about 66 (18%) respondents had positive attitude about family planning and more than half 300 (82%) had negative attitude as shown in Table 4.

**Table 4:** attitude of respondents on modern contraceptive methods in Durame District, Southern Ethiopia, 2021 (N=366)

Variables	Frequency	Percentage
**What do you think about family planning**		
Important	292	79.8
Not important	23	6.3
I don't know	51	13.9
**Insertion and removal of implant is highly painful**		
Agree	64	17.5
Disagree	23	6.3
Not sure	279	76.2
**Insertion of IUCD cause to lose privacy**		
Agree	64	17.5
Disagree	21	5.7
Not sure	281	76.8
**Use of IUCD restrict normal activities**		
Agree	65	17.8
Disagree	18	4.9
Not sure	283	77.3
**Operation for female sterilization is dangerous**		
Agree	112	30.6
Disagree	18	4.9
Not sure	236	64.5
**Irregular bleeding is severe after implant use**		
Agree	83	22.7
Disagree	21	5.7
Not sure	262	71.6
**Operation for male sterilization is unacceptable**		
Agree	140	38.3
Disagree	19	5.2
Not sure	207	56.5

IUCD: intra-uterine contraceptive device

**Community level barrier by respondents to use family planning:** the main community level barriers that hindered from using family planning by respondents were cultural issues 139 (38%) followed by fear of infertility, fear of developing side effects, lack of knowledge, need of more children and due to religion, 80 (21.9%), 20.5%, 8.7%, 6% and 4.9% respectively.

**Institutional level barrier by respondents to use family planning:** the major institutional level barriers that prevent from using family planning by respondents were lack of male provider in the health facilities 154 (42.1%) followed by non-availability of service 90 (24.3%), Insufficient availability of male-friendly services 51 (13.9%), lack of time 42 (11.5%) and negative provider attitude 30 (8.2%).

**Policy level barrier to use family planning by respondents:** the key policy level barriers that avert from using family planning by respondents were poor support for policies and programmers focused on male involvement in family planning 132 (36.1%) followed by absence of male involvement strategies in the county integrated development plans 87 (23.8%), absence of social and behavior change interventions 75 (20.5%), lack guidance on how to share decision-making while negotiating choices with their partners 39 (10.7%), inadequate implementation of the comprehensive approach to men participation in family planning services 28 (7.7%) and unavailability of health budgets, services and products for men 5 (1.4%).

**Factors associated with men's involvement in family planning:** in the bivariate logistic regression analysis, men's involvement in family planning was significantly associated with, educational status of married men, number of currently living children, respondents´ knowledge, sources of information about FP and attitude of the study subjects were met the minimum criteria (P <0.25) for further multivariate logistic analysis. Table 5 shows the multivariate logistic regression analysis; men's involvement in family planning was significantly associated with educational status of the respondent, number of currently living children, source of information, knowledge and attitude of the respondents.

**Table 5:** factors associated with men's involvement in family planning in Durame Town, Southern Ethiopia, 2021

Variables		Men's involvement in family planning		COR (95%CI)	AOR (95%CI)
		Yes	No		
Educational status of the respondent	Illiterate	36	0	-	-
	Can read and write	16	95	3.49 (1.98, 6.13) a	2.97 (1.46, 6.04) b
	Primary and secondary education	55	36	0.38 (0.2, 0.74) a	0.72 (0.05, 0.31)b
	Diploma and above	39	89	1	
Number of currently living children	1-2	159	79	1	
	3-4	39	42	2.17 (1.29, 3.62) a	3.76 (1.57,8.96)b
	≥ 5	22	25	2.28 (1.22,4.31) a	6.93 (0.38,2.65)
Source of information	Health institution	18	51	1.9 (0.41, 2.87)	1.71 (0.42, 6.92)
	Friends	17	46	0.95 (0.35, 2.59)	1.54 (0.35, 6.84)
	Media	102	94	4.24 (1.85,9.71) a	6.71 (1.99, 22.4)b
	NGO	9	29	1	
Knowledge on modern family planning methods	Adequate knowledge	95	106	2.14 (1.39, 3.3) a	2.58 (1.38, 4.85) b
	In adequate knowledge	51	114	1	
Attitude of respondents	Negative attitude	14	206	1	
	Positive attitude	30	116	3.81 (1.94,7.47)a	10.9 (4.3,27.8)b

a: statistically significant in COR: P-value <0.25; b: statistically significant in AOR: P value <0.05; AOR: adjusted odds ratio; COR: crude odds ratio

The finding of multivariable logistic regression analysis revealed that educational status of the participants was significantly associated with the involvement in family planning, in that respondents who can read and write were 2.97 times more likely to involve in modern family planning utilization compared to those who have diploma and above education (AOR: 2.97, 95 % CI: 1.46, 6.04). Besides, participants whose levels of education primary and secondary were 28% less likely to participate in modern family planning compared to participants who have diploma and above. Statistical association also depicted that number of currently living children was significantly associated with the out-come variable, in that, couples who had 3-4 children were 3.76 times more likely to use modern family planning than couples who had 1-2 children (AOR: 3.76, 95% CI: 1.57, 8.96).

Moreover, those men who heard media messages on contraception in the last 1 year were 6.71 times more likely to involve modern contraception, when it is compared with those who were not exposed to such messages (AOR = 6.71, 95% CI: 1.99, 22.4). Knowledge on modern family planning was also significantly associated with male involvement in family planning, as, participants who had adequate knowledge were 2.58 times more likely to involve in contraception compared to those who have inadequate knowledge on modern family planning methods (AOR = 2.58, 95% CI: 1.38, 4.85). Respondents with positive attitude towards modern contraception was 10.9 times more likely involvement in contraception than respondents who had negative attitude towards involvement in contraception (AOR= 10.9, 95% CI: 4.3, 27.8).

## Discussion

Engaging men actively and acquiring their support to family planning is a significant predictor of the likelihood for family planning service utilization. In current study, the prevalence of married men's involvement in family planning was 69.7% which is in line with the study done in Arba Minch and Debre Tabor towns, 68% [21] and 68.1% [22] respectively. The present study was higher than the studies conducted in Wolaita Sodo Town 60% [23], Hosanna Town 48% [24] in Kenya 52% [18], Turkey 30% [25], in West Shewa Zone, Ethiopia 36% [26]. This discrepancy may be due to the time difference in this study and the socio-cultural difference of the community. The other justification might be governmental concern, individual motivation and conversation and support of the service use by male partners. The result of the study is lower than the study conducted in Vietnam 74.4% [4]. The reason may be due to socio-demographic variation between the two countries and accesses to family planning methods.

The results of this study found that only 9.8% of men were involved on their own by using family planning methods. The result of current study is higher than the studies done in, Debre Tabor 7.5% [21], Wolaita Sodo 5% [23], Debre Marcos 8.4% [9], West Pokot, Kenya 6% [18]. On the other hand, the findings of present study lower than Eastern Tigray, Ethiopia 15% [27], Uganda, 40.0% [28] and India 71.0% [29]. Amazingly, none of the study subjects used permanent methods (sterilization) themselves. Only 10% of males used condoms during study period; this suggesting that the practicing of other permanent methods was remarkably reduced. This might be linked to the limited choice of accessibility of male contraceptives and partly because the cultural norms against male sterilization in the rural community. Hence then, working towards publicizing permanent family planning for those whose ideal number of children should be built up.

In the current study male participation was as well measured by spousal communication. The finding of this study revealed about 45% of study participants were discussed with their wives. This result is consistent with the studies conducted in south eastern zone of Tigray [27], Harar, Eastern Ethiopia [30], Angolela Tera District [31], Womberma District, Northern Ethiopia [32] a rural community of Western Ethiopia [33] in Bangladesh [34], in Angecha Woreda, Kembata Tembaro Zone [35] and in rural areas of Akwa Ibom State, Nigeria [36] had showed that those who communicate with their wives were more predisposed towards use of contraceptive methods. In the present study almost 60% of men supported their wives, which is in line with the study conducted Womberma District, Northern Ethiopia [32], but lower than other study findings in Hossana Town, Southern Ethiopia [24] and eastern zone of Tigray, Ethiopia [27]. The difference might be explained by cultural differences between the communities.

In current survey, none of the study subjects used permanent methods (sterilization) by themselves and only 10% of males used condoms during study period; which is lower than the finding of Loka Abaya District [37] but higher than Wolaita Sodo Town [23] and Hosanna Town [24]. Yet there is improvement in male participation on use of family planning method; the study showed lower practice on contraceptive methods that could be used by men. This study suggests that practice of males with regard to family planning need special concentrations to space and limit the number of children by scaling up forms and practice of contraceptive methods. According to the respondents, the reason for not using family planning methods in the study area might be community level, institutional level and policy level barriers. It is consistent with the studies done in Republic of Kenya [5] West Pokot County, Kenya [18], Nigeria [38], developing countries [39] and Eastern Nepal [40].

According to the analytic part of this study, educational status of the respondent, number of currently living children, source of information, knowledge and attitude of the respondents were statistically significant predictors of utilization of contraceptive methods. The result highlighted that educational status of male partner was found to be one factor for men's involvement in family planning; those respondents who can read and write were 2.97 times more likely to involve in modern family planning utilization compared to those who have diploma and above education level (AOR: 2.97, 95% CI: 1.46, 6.04). This variable was not significantly associated with other studies conducted previously. Besides, respondents who have educational level of primary and secondary were 28% less likely to involve in modern family planning utilization compared to those who have diploma and above education level (AOR: 0.72, 95% CI: 0.05, 0.31). This study result is lower than the finding of Arba Minch Town [21]. This may be due to lack of information about family planning from different corner and accesses to family planning methods.

This study revealed that number of currently living children was significantly associated with the out-come variable (AOR: 3.76, 95% CI: 1.57, 8.96). This finding is in line with the studies conducted in different countries [11,18,38,41-44]. Possible explanations for this could be that those with larger families could have achieved the number of children they wanted to have, which implies that they use methods to limit further child birth. The study also showed, that men who heard media messages on family planning in the last 12 months were 6.71 times more likely to involve modern family planning utilization, when it is compared with those who were not exposed to such messages (AOR=6.71, 95% CI: 1.99, 22.4). The study is consistent with the finding of Mizan-Aman District, Southwestern Ethiopia [8], in, Debre Tabor [22], in Bangladesh [34] and urban Senegal [45].

Moreover, the result of the study showed that respondents who had adequate knowledge were 2.58 times more likely to involve in family planning utilization compared to those who have inadequate knowledge on modern family planning methods (AOR=2.58, 95% CI: 1.38, 4.85). It is consistent with the studies done in Debre Markos, Arba Minch, Tigray, Gedeo Zone, Bahir Dar and Afar, South West Region: Cameroon and Malegedo Town West Shoa Zone, Oromia, Ethiopia [9,21,27,46-50]. Furthermore, participants having positive attitude towards contraception were 10.9 times more likely to be involved family planning service utilization than those having negative attitude. (AOR=10.9, 95% CI: 4.3, 27.8). The finding of current study is consistent with the results of the previous studies; Nigeria, India and Gedeo Zone [46,51,52].

## Conclusion

The study recommends that the prevalence of male participation in contraception in the study area is yet inadequate despite interventions government and nongovernmental organizations. Hence, more efforts should be made to improve education standards particularly for men in terms provision of training on contraception to make them more available to men. All responsible bodies must confirm accessibility, approachability and maintainable promotion for contraceptive services. The contraceptive programs should integrate males in the uptake of family planning services.

**Funding:** the authors received financial support from Wachemo University Durame Campus for the research.

### What is known about this topic

- Males dominate in taking important decisions in the family, including reproduction, family size and contraceptive use;
- Family planning is very crucial in terms of decreasing maternal mortality, reducing poverty and environmental degradation;
- Male involvement is important in sharing responsibility in reproductive health (RH) matters like; controlling STIs including HIV/AIDS and adolescent gestations.

### What this study adds

- This is the first study to the area and identified the prevalence male participation on family planning utilization;
- Men's involvement in family planning was significantly associated with educational, number of currently living children, source of information, knowledge and attitude of the respondents;
- Male involvement in family planning is low due to individual, facility, community and policy level reasons.
